# Evaluation of cross-cultural adaptation and validation of the Persian version of the critical thinking disposition scale: methodological study

**DOI:** 10.1186/s12912-024-02129-y

**Published:** 2024-07-08

**Authors:** Hossein Bakhtiari-Dovvombaygi, Kosar Pourhasan, Zahra Rahmaty, Akbar Zare-Kaseb, Abbas Abbaszadeh, Amirreza Rashtbarzadeh, Fariba Borhani

**Affiliations:** 1 Student Research Committee, School of Nursing and Midwifery, Shahid Beheshti University of Medical Sciences, Tehran, Iran; 2 Medical Ethics and Law Research Center, Shahid Beheshti University of Medical Sciences, Tehran, Iran; 3 Institute of Higher Education and Research in Healthcare, Department of Biology and Medicine, Lausanne University Hospital and University of Lausanne, Lausanne, Switzerland; 4 School of Nursing & Midwifery, Shahid Beheshti University of Medical Sciences, Tehran, Iran

**Keywords:** Critical thinking, Nursing student, Psychometric properties, Validity, Reliability

## Abstract

**Introduction:**

Assessing critical thinking disposition is crucial in nursing education to foster analytical skills essential for effective healthcare practice. This study aimed to evaluate the cross-cultural adaptation and validation of the Persian version of the Critical Thinking Disposition Scale among Iranian nursing students.

**Method:**

A total of 390 nursing students (mean age = 21.74 (2.1) years; 64% female) participated in the study. Face and content validity were established through feedback from nursing students and expert specialists, respectively. Construct validity was assessed using exploratory factor analysis (EFA) and confirmatory factor analysis (CFA). The EFA was used to explore the number of factors and the items that were loading on them. The CFA was used to confirmed the fidnings of the EFA on the same sample. Convergent and discriminant validity were examined, along with reliability through internal consistency and test-retest reliability.

**Results:**

EFA revealed a two-factor structure, comprising “Critical Openness” and “Reflective Skepticism,” explaining 55% of the total variance. CFA confirmed the model’s fit (χ² = 117.37, df = 43, χ²/df = 2.73, *p* < 0.001; RMSEA = 0.067; CFI = 0.95; TLI = 0.93, SRMR = 0.041). Convergent and discriminant validity were supported, with significant factor loadings (*p* < 0.001) ranging from 0.61 to 0.77. The CTDS exhibited strong internal consistency (α = 0.87) and excellent test-retest reliability (ICC = 0.96).

**Conclusion:**

The validation of the CTDS in Persian language settings provides a reliable tool for assessing critical thinking disposition among Iranian nursing students. The two-factor structure aligns with previous research, reflecting students’ propensity towards critical openness and reflective skepticism. The study’s findings underscore the importance of nurturing critical thinking skills in nursing education.

## Introduction

Critical thinking can be seen as the ability to think logically, dynamically, comprehensively and practically when judging a situation to investigate and make appropriate decisions [1, 2]. This ability helps to gain insight and examine an idea or concept from different perspectives [3]. Critical thinking has become an educational ideal, with most policy makers and educationists calling for the development of critical attitudes in students [4]. Critical thinking has been identified as one of the most important outcomes of higher education courses [5].

There is increasing evidence showing that critical thinking is considered an important part of preregistered nursing students and registered nurses when they are working in various clinical practice settings [6, 7]. Critical thinking is one of the basic skills that prepares nursing students to effectively manage patient problems, make the best clinical decisions, provide safe and high-quality care, and better control critical situations. On the other hand, negative consequences such as depression, failure to solve patient problems, and incomplete clinical reasoning can be consequences of poor critical thinking [8, 9].

Critical thinking is expected in nursing program graduates at the international level [10, 11]. Therefore, it is important to evaluate and measure the levels of critical thinking of nursing students at different stages and education so that educators can adjust learning activities to ensure the desired results [2, 12, 13]. Educators are the ones who are responsible for and have the opportunity to shape this skill during the years of education and trayning new generations [14]. Despite the importance of critical thinking in the nursing profession, studies have reported a lack of critical thinking skills among undergraduate students in the field [15, 16].

Critical thinking has two main components: critical thinking skills and critical thinking disposition (CTD). The skills component refers to the cognitive processes of thinking, while the disposition component refers to personal desire and internal motivation for critical thinking [9]. Several studies have highlighted the need for reliable assessment tools for critical thinking, specifically in nursing, rather than in a general context [17–19].

To our knowledge, and based on our literature review, the CTD is the only specific tool for assessing the tendency to think critically. However, this tool has not been used or validated in the Iranian educational context, population and language. Considering the lack of effective tools for evaluating CTD in undergraduate nursing programs in Iran, the purpose of this study was to translate and evaluate the psychometric properties of the Persian version of CTD among nursing students.

## Methods

### Study design

This was a cross-sectional study utilizing cross-cultural adaptation to translate and investigate the validity and reliability of the CTDS for use among Iranian nursing students [20]. The translated scale underwent examination for reliability and validity tests.

### Study population and sampling

Convenience sampling was employed at the School of Nursing, Shahid Beheshti University in Tehran. This method involved selecting participants who were readily available and willing to take part in the study. Specifically, the study targeted all undergraduate nursing students, who were invited to participate in the research. Recruitment continued until the desired sample size was achieved. To maintain the integrity of the data, students who submitted incomplete questionnaires were excluded from the analysis. Undergradute studies in Iran normally involve 4 years of education in general nursing, as well as clinical rotations in all hospital units and public health sectors.

There are two general recommendations concerning the minimum sample size necessary for conducting factorial analysis. The first recommendation emphasizes the significance of the absolute number of cases (N), while the second recommendation highlights the importance of the subject-to-variable ratio (p). Guilford suggested that N should be no less than 200 [21]. Additionally, MacCallum et al. recommended that the subject-to-variable ratio should be at least 5 [22]. A total of 390 nursing students voluntarily participated in the study.

### Measurements

The CTDS, developed by Sosu, is an instrument used to measure the dispositional dimension of critical thinking [23]. Self-report questionnaires were given to the students. The demographic questionnaire collected included participants’ age, gender, education, and grade point average (GPA). The Critical Thinking Disposition Scale (CTDS) was used to measure the dispositional aspect of critical thinking. This scale comprises 11 items, employing a five-point Likert-type response format (1 = strongly disagree; 2 = disagree; 3 = neutral; 4 = agree; 5 = strongly agree). Total scores range from 11 to 55. The subscores include the first seven items reflecting a level of critical openness with a score ranging from 7 to 35 and the last four items indicating a level of reflective skepticism with a score ranging from 4 to 20. Higher CTDS scores indicate a greater degree of critical thinking [23].

### Translation of the CTD scale

Following correspondence with the instroment developer, Dr. Sosu, and obtaining permission, the scale underwent translation using the standard Backward-Forward method. Initially, the scale was independently and simultaneously translated from English to Persian by two translators proficient in both Farsi and English. In the subsequent phase, these translations were juxtaposed and merged into a unified translation. This facilitated the comparison and identification of discrepancies, which were then rectified based on feedback from a panel of experts, including two psychometric experts and two nursing professors. In the third stage, the resulting Persian version was given to two translators fluent in Persian and English (distinct from those in the initial experts) to translate it back to English, thereby completing the translation of the scale from Persian to English. In the fourth stage, the two English translations were compared, and any disparities were resolved by the experts, culminating in a single translation. Subsequently, the prefinal version was evaluated for content and face validity.

### Face validity

Face validity refers to the degree to which a measurement method seems to accurately measure the intended construct [24]. In a qualitative assessment of face validity, 10 nursing students were asked to evaluate factors such as the clarity of phrases and words, the coherence and relevance of items, the potential for ambiguity, and the necessity of removing or combining items. Additionally, two nursing professors and two psychometric specialists scrutinized the scale to determine whether it indeed appeared to measure its intended construct.

### Content validity

Content validity examines the extent to which a collection of scale items aligns with the pertinent content domain of the construct it aims to assess [24]. The qualitative evaluation of the content validity involved a panel consisting of two nursing professors in the field of nursing education and two statisticians who were experts in psychometric topics. Their input on item placement, word selection, grammar adherence, and scoring accuracy of the scale and its instructions were solicited, with their feedback serving as the foundation for any required adjustments.

## Construct validity

### Exploratory factor analysis

To explore the number of existing subscales and potential factors, Exploratory Factor Analysis (EFA) was performed using Principal Component Analysis (PCA) with varimax rotation. The scree plot and parallel analysis suggested the number of existing factors. The scree plot displays the eigenvalues of each factor extracted from the data in descending order. The number of factors was retained by examining the slope of the curve. A sharply decreasing slope indicates the optimal number of factors that capture the most variance in the data.

### Confirmatory factor analysis

To confirm the findings of the EFA, Confirmatory Factor Analysis (CFA) was conducted using MPLUS to confirm the 2-factor structure identified with the items loaded on each factor in the EFA. Model fit indices, including Comparative Fit Index (CFI), the standardized residual root mean squared error (SRMR) of approximation and the root mean squared error of approximation (RMSEA), with cutoff points of > 0.95, < 0.08 and < 0.06, respectively, were used [25]. Factor loadings were reported using standardized beta coefficients to evaluate the strength of the relationships between items and factors, and a *p* value of 0.05 was considered a significant factor loading.

### Convergent and discriminant validity

The mean scores for Critical Openness and Reflective Skepticism were computed. Convergent and Discriminant Validity was checked for correlations between students’ GPA as an indicator of academic achievement and the scores of the subscales of the CTDS.

### Reliability

To assess reliability, internal consistency was tested using Cronbach’s alpha coefficient calculations for the total score and subscores. To assess the consistency of the test-retest approach over a two-week period, a group of 40 individuals from the target demographic underwent examination. Their scores from both sessions were analyzed to determine test-retest reliability, and the Intraclass Correlation Coefficient (ICC) was calculated.

### Ethical considerations

The current study underwent assessment and received approval from the Research Ethics Committee at Shahid Beheshti University of Medical Sciences (Ethical code: IR.SBMU.RETECH.REC.1403.013). Permissions were duly acquired from the pertinent authorities at the research sites as well as the developer of the original scale. Nursing students were provided with comprehensive information regarding the study’s objectives, their right to withdraw from participation, and the confidentiality of their data. Informed consent was obtained from all participating students. All procedures adhered strictly to the appropriate guidelines and regulations.

### Data analysis

Descriptive statistics, including the mean, standard deviation, median, range, and frequency, were used to describe the population and their critical thinking scores. Analysis of demographic characteristics, EFA, and reliability tests were performed using IBM SPSS Statistics (Version 27). CFA was performed using MPLUS (Program Copyright © 1998–2017 Muthén & Muthén Version 8) software.

## Results

### Characteristics of the participants

A total of 390 participants (mean age = 21.74 (2.1) years; 64% female) completed the questionnaire.

### Face validity and content validity

Face validity was established through the feedback of 10 nursing students, while content validity was assessed by four expert specialists. No alterations were made to the items in terms of their simplicity and clarity during the evaluation of both face and content validity.

## Construct validity

### Exploratory factor analysis

**Fig. 1:**
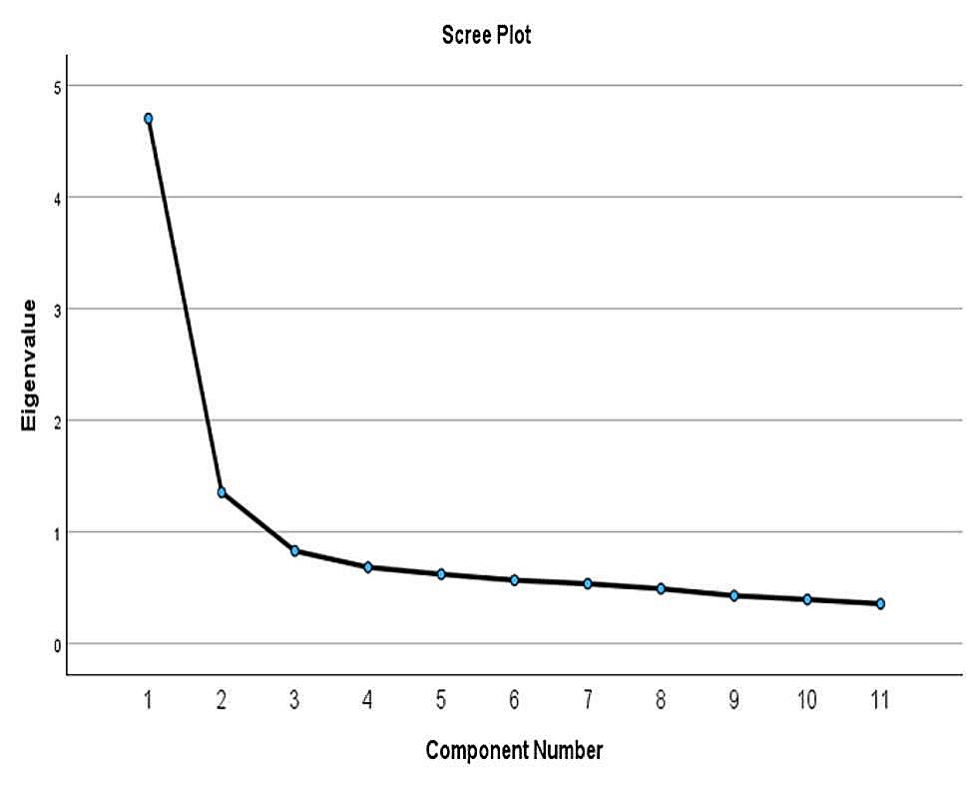
Scree Plot of the Persian version of the Critical Thinking Disposition Scale

The scree plot and parallel analysis suggested a two-factor solution (Fig. 1), which accounted for 55% of the total variance in the scores (Table 1). Factor loadings revealed a clear factor structure, with items loading on two factors. The rotated factor loadings are presented in Table 2. The items clustered together on two distinct factors, with no cross-loadings observed.

**Table 1:** Total Variance explained

Component	Initial Eigenvalues			Extraction Sums of Squared Loadings			Rotation Sums of Squared Loadings		
	Total	% of Variance	Cumulative %	Total	% of Variance	Cumulative %	Total	% of Variance	Cumulative %
1	4.707	42.793	42.793	4.707	42.793	42.793	3.194	29.032	29.032
2	1.358	12.347	55.141	1.358	12.347	55.141	2.872	26.109	55.141
3	0.833	7.570	62.710						
4	0.686	6.239	68.949						
5	0.624	5.671	74.620						
6	0.570	5.184	79.804						
7	0.539	4.898	84.702						
8	0.494	4.491	89.193						
9	0.432	3.925	93.118						
10	0.398	3.614	96.733						
11	0.359	3.267	100.000						

Extraction Method: Principal Component Analysis.

**Table 2:** Rotated Component Matrix^a^

Items	Component	
	1	2
Item 1	0.556	0.426
Item 2	0.738	0.146
Item 3	0.674	0.182
Item 4	0.704	0.227
Item 5	0.707	0.109
Item 6	0.599	0.329
Item 7	0.616	0.334
Item 8	0.149	0.769
Item 9	0.102	0.765
Item 10	0.270	0.750
Item 11	0.209	0.783

Extraction Method: Principal Component Analysis.

Rotation Method: Varimax without Kaiser Normalization.

a. Rotation converged in 3 iterations.

The two factors were interpreted as follows: Factor 1, labeled “Critical Openness,” comprised items related to the level of critical openness; Factor 2, labeled “Reflective skepticism” included items reflecting the level of reflective skepticism. These factors align well with previously established dimensions.

### Confirmatory factor analysis

CFA confirmed the model including the two factors with their respective indicators based on the EFA results. The CFA model demonstrated acceptable fit to the data: χ² (55) = 1500.38, *p* < 0.001; RMSEA = 0.067; CFI = 0.95; TLI = 0.93, SRMR = 0.041. Although the chi-square test was significant, other fit indices indicated a reasonably good fit to the data.

**Fig. 2:**
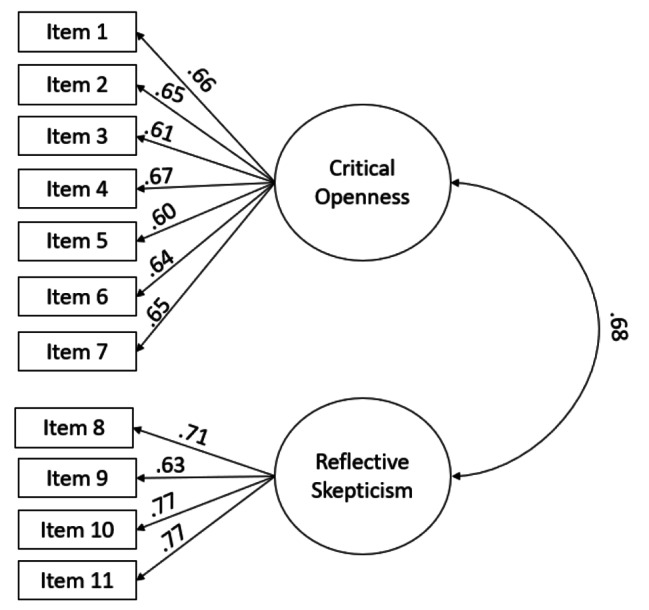
Factor structure of the Persian version of the Critical Thinking Disposition Scale

The standardized factor loadings ranged from 0.61 to 0.77, Fig. 2, all of which were statistically significant (*p* < 0.001) (Table 3). These loadings provided further support for the factor structure identified in the EFA.

**Table 3:** Standardized factor loading using confirmatory factor analysis

	Estimate	S.E	Est./S.E	*P*-Value
Reflective skepticism				
Item 8	0.671	0.034	19.450	< 0.001
Item 9	0.634	0.037	17.334	< 0.001
Item 10	0.767	0.029	26.858	< 0.001
Item 11	0.774	0.028	27.584	< 0.001
Critical Openness				
Item 1	0.660	0.034	19.233	< 0.001
Item 2	0.654	0.035	18.790	< 0.001
Item 3	0.612	0.037	16.549	< 0.001
Item 4	0.669	0.034	19.735	< 0.001
Item 5	0.602	0.037	16.103	< 0.001
Item 6	0.640	0.036	18.017	< 0.001
Item 7	0.654	0.034	19.026	< 0.001

### Convergent and discriminant validity

Convergent and discriminant validity were examined by determining factor scores based on item allocation. The analysis revealed a sample mean of 28.65 (SD = 2.7) for the Critical Openness factor and a mean of 16.8 (SD = 1.8) for Reflective Skepticism. A weak yet statistically significant correlation was observed between students’ GPA and their level of critical openness (*r* = 0.15, *p* = 0.003), indicating a slight association between academic performance and this aspect of cognitive disposition.

### Reliability

The reliability of the CTDS-P was assessed through rigorous statistical analysis. Internal consistency was robust, as indicated by a Cronbach’s alpha coefficient of 0.87 for the overall scale, demonstrating the coherence of the items within the measure. Subscale analysis revealed strong reliability, with values of 0.83 for critical openness and 0.80 for reflective skepticism, indicating the consistency of responses across different dimensions of the construct. Additionally, the scale exhibited excellent test-retest reliability, as evidenced by an Intraclass Correlation Coefficient (ICC) of 0.96, with a 95% confidence interval ranging from 0.93 to 0.98, suggesting high stability and consistency of the scores over time.

## Discussion

The CTDS has undergone translation and cross-validation in different populations across the USA [26], Norway [27], Brazil [28], Spain [29], Turkey [30], and Vietnam [31]. The reliability and validity of this scale have been demonstrated in studies conducted among high school students [29] and university students [26–28, 30, 31]. The CTDS was first introduced as a tool to measure critical thinking disposition in undergraduate and postgraduate students [4].

This study used comprehensive reliability and validity tests to validate the CTDS in the Persian language and in Iranian nursing student participation. This study revealed the existence of two factors, critical openness and reflective skepticism. These factors align well with previously established studies [4, 27, 30, 31]. Conversely, Spanish, Brazilian, and US versions demonstrated that the one-factor model fit better for their population [26, 28, 29].

### Validity

In the face validity and content validity tests, neither the simplicity nor the clarity of the items were altered. The validity of the content was done qualitatively. Similar to previous quantitatively measured studies, our study has also confirmed the validity of the content [28, 31].

### Reliability

The internal consistency of the CTDS demonstrated the coherence of the items within the measure. Several studies have reported similar internal consistency values for the CTDS, with Cronbach’s alpha measuring 0.88 according to Nguyen et al. 2023. Sosu et al. 2013, Akin et al. 2015, Yockey 2016, Bravo et al. 2020, and Gerdts-Andresen et al. 2022 also reported values of 0.79, 0.78, 0.79, 0.77, and 0.76, respectively. It is widely recognized that a Cronbach’s alpha coefficient of 0.70 or higher is acceptable [32]. Consequently, the CTDS has exhibited strong internal consistency across diverse linguistic and cultural contexts. In addition, the scale exhibited excellent test-retest reliability, thereby indicating a high level of stability and consistency in scores across time. Additionally, our study demonstrated an outstanding ICC [33, 34].

### Limitaion

There are several limitations to this study. Initially, the self-assessment survey may have been prone to social desirability bias, leading to potential overestimation of reported measures. To mitigate bias, this study utilized an anonymous survey. Moreover, the study used a cross-sectional design, which prevented the establishment of prospective predictive validity.

## Conclusion

To conclude, our investigation establishes the CTDS as a reliable and valid tool for evaluating critical thinking disposition among Iranian nursing students. With its two-factor structure of “Critical Openness” and “Reflective Skepticism,” the scale offers valuable insights into cognitive disposition. Its robust psychometric properties underscore its potential for enhancing critical thinking skills in healthcare education and practice. Further research avenues may explore its nuanced applications in fostering analytical reasoning and problem-solving abilities.

## Data Availability

The datasets generated and/or analysed during the current study are not publicly available due to the necessity to ensure participant confdentiality policies and laws of the country but are available from the corresponding author on reasonable request.
